# Prevalence and associated factors for asymptomatic microscopic hematuria in adults in the PERSIAN Guilan cohort study (PGCS)

**DOI:** 10.1038/s41598-024-53597-w

**Published:** 2024-02-11

**Authors:** Farahnaz Joukar, Soheil Hassanipour, Amirhomayoun Atefi, Saman Maroufizadeh, Ardalan Akhavan, Mohammadreza Naghipour, Siavash Falahatkar, Mohammad-Javad Khosousi, Mehrnaz Asgharnezhad, Fariborz Mansour-Ghanaei

**Affiliations:** 1 Gastrointestinal and Liver Diseases Research Center, Guilan University of Medical Sciences, Rasht, Iran; 2 Department of Biostatistics and Epidemiology, School of Health, Guilan University of Medical Sciences, Rasht, Iran; 3 Urology Research Center, Guilan University of Medical Sciences, Rasht, Iran

**Keywords:** Risk factors, Signs and symptoms, Urology

## Abstract

Although hematuria is not life-threatening, some could be the result of a more severe condition. Our objectives are to report on the prevalence and risk factors of asymptomatic microscopic hematuria (AMH) in the prospective epidemiological research studies of the Iranian adults (PERSIAN) Guilan cohort study (PGCS) population. This cross-sectional study was conducted from 2014 to 2017 and consisted of 10,520 individuals aged 35–70. Data collection was conducted using a questionnaire during a face-to-face interview. The urine analyses (UA) were done up to 2 h after sample collection. Based on a urine microscopy evaluation, AMH is defined as 3 or more red blood cells per high power field (HPF). Simple and multiple logistic regression analysis was conducted to explore factors associated with AMH. The prevalence of AMH in this study was 34.1% and was more prevalent in participants of older ages and female gender as well as those with low educational level, underweight-body mass index (BMI), high physical activity, smoking, alcohol consumption, and kidney stone disease. On the other hand, obesity, opium, and diabetes decreased the likelihood of AMH. The results of the present study shed light on the prevalence and risk factors of AMH and suggested that a significant portion of the study population is affected by AMH. Considering the lack of consensus on a definite clinical guideline for AMH in our country, the results of the present study could be used to design a unit algorithm for screening and therapy of AMH.

## Introduction

Hematuria is the presence of red blood cells (RBCs) in the urine, usually considered a symptom of benign or malignant kidney diseases or urogenital tract damage^1^. If this phenomenon causes a visible color change in urine, it is called gross hematuria^1^. In contrast, the definition for asymptomatic microscopic hematuria (AMH) is the presence of 3 or more RBCs per high power field (HPF) on an adequately collected microscopic urinalysis as per the American Urological Association guidelines^2^. Hematuria is often asymptomatic, seen in 4–5% of routine urinalysis^1^. Hematuria is a common symptom of kidney damage in 80–94% of cases^3^. A variety of etiologies has been described for microscopic hematuria, such as immunoglobulin A nephropathy, thin basement membrane disease, Alport syndrome, and benign prostate hyperplasia. Hematuria can also result from infections, urolithiasis, malignancy, or other lesions that may obstruct the urinary tract, raise intra-renal pressures, and negatively affect kidney function^2,4–6^.

Further studies indicated that AMH with protein excretion increases the chance of an important disease, so screening for hematuria and proteinuria may have benefits in prevention, delaying the progression of disease, and reducing the number of progressive renal failures^7,8^. Correspondingly, several studies have shown that hematuria is one of the most common signs and symptoms of bladder cancer^9–11^. The 2020 American Urological Association **(**AUA**)** guideline recommends assessing risk factors for genitourinary malignancy, medical renal disease, and gynecologic and non-malignant genitourinary causes of microhematuria^2^. In Iran, many studies have been performed to determine the prevalence of AMH in different diseases, and they all suggested AMH as a significant sign for probable renal diseases and urothelial tumors that should be followed up with a standard diagnostic approach^12–15^. Unlike proteinuria, there is still much controversy on the natural course and clinical implications of AMH. Thus, earlier diagnosis of underlying disease and improved patient survival could be achieved by prompt evaluation of this condition. However, the cost-effectiveness of mass screening for AMH in the general population is still questionable. Previous research suggested its benefit in populations with AMH as a common finding^16^. Because of the lack of sufficient high-quality evidence regarding determinants and clinical course of hematuria, especially AMH, there are no consistent guideline recommendations for screening and therapy of AMH. Determining the factors associated with AMH could specify target populations that should be a priority for AMH screening and therapeutic programs. Also, these relations between AMH and its associated factors could serve as a target for further studies delineating the condition’s pathophysiology. Our objectives are to report on the prevalence and determinants of AMH in the Guilan adult cohort population.

## Methods

### Location and patients

This observational study used the data from the PERSIAN Guilan Cohort Study (PGCS) as a part of the PERSIAN (Prospective Epidemiological Research Studies in IRAN) cohort^17,18^ with a sample size of 10,520 males and females aged between 35 and 70 years in Some’e Sara County (including 39 villages and urban regions), Guilan, Iran^19^. This cohort study aimed to investigate the prevalence and incidence of non-communicable diseases and improve this province’s lifestyle. The investigation protocol of the PGCS, as well as the process of lab measurements, sampling, and physical examinations, is available online^19^. Data collection (clinical and demographic characteristics, blood pressure anthropometric measurements, comorbidities, laboratory, and spirometry tests) was conducted according to the study protocol from 2014 to 2017. The convenience sampling method was adopted to choose the study population. Geographical location of participants was determined using Garmin GPSMAP 78s and the map was generated by ArcGIS software (Version 10.8 for Desktop).

### Data collection and measurements

Data were obtained from the participants during a face-to-face interview using a questionnaire of the participants’ demographic characteristics and determinants of AMH. Collected data are listed as follows: demographic variables, including gender (female, male), age, and habitat (rural, urban); and socioeconomic information, including education level (years), marital status (single, married, divorced, and widow), employment status (employed or unemployed), and wealth score index (WSI). WSI was reported using the principal component analysis (PCA)^20^. Detailed explanations of the WSI calculation for participants were included in past studies from the PERSIAN cohort^20,21^. The present study categorizes WSI into three tertile, from poorest (1st tertile) to most affluent (3rd tertile)^20^.

Individual factors, including body mass index (BMI), smoking status (yes, no), hookah smoking (yes, no), ever opium consumption (yes, no), ever alcohol use (yes, no), and physical activity. Regarding the individual factors, the participants were categorized according to their BMI (average weight, underweight, overweight, and obese for BMI levels of 18.5–24.9 kg/m^2^, < 18.5 kg/m^2^, 25–29.9 kg/m^2^ and 30 kg/m^2^, respectively)^22^. Physical activity previously described in detail in past studies from PERSIAN cohort^20,23^ was measured using metabolic equivalent rates (METs), a self-report instrument for measuring the activities of daily living^24^ of participants of PERSIAN cohort using the questionnaire.

Past medical history including hypertension (HTN), diabetes, and kidney stone disease (yes, no). Concerning past medical history, diabetes was characterized as using insulin or glucose-lowering drugs, fasting plasma glucose of ≥ 126 mg/dL, or a self-report of diagnosis of diabetes by a physician. Hypertension was characterized as taking blood pressure-lowering drugs, a systolic blood pressure > 140 mmHg, diastolic blood pressure higher than 90 mmHg, or a self-reported diagnosis of hypertension by a physician^19^.

According to the PGCS protocol for each participant, at least 10 mL of morning urine samples were collected and labeled by trained technicians. The urine analyses (UA) were done up to two hours after sample collection. Microscopic evaluation of the samples was conducted on the sediment prepared after centrifugation of the samples at 1000 rpm for 10 min. In the recent study, AMH was defined as three or more red blood cells per HPF on an adequately collected microscopic urinalysis according to the American Urological Association guidelines^2^. Urine samples of female patients were collected beyond the first week of menstrual period. Due to the higher-than-expected prevalence of AMH in the primary analysis of the first 1000 patients, a second independent laboratory double-checked one per every 100 urine samples during the rest of the study. The similarity rate between the results of the two laboratories was about 95%.

### Statistical analysis

This study presented continuous variables as mean ± standard deviation (SD) and categorical variables as number (percentage). We used the chi-square test (or Cochran—Armitage test for trend) for categorical data to test for differences between those with and without hematuria. Simple and multiple logistic regression analysis was conducted to explore factors associated with hematuria. The crude and adjusted odds ratio (OR) and 95% confidence interval (CI) were calculated. In addition, multiple logistic regression analyses were used to estimate the OR of hematuria by gender. In all multiple regression analyses we used the backward elimination method (alpha to remove = 0.05). Statistical analysis was done with SPSS for Windows, version 16.0 (SPSS Inc., Chicago, IL, USA), and the significance level was set at 0.05.

### Ethics approval and consent to participate

The Helsinki Declaration and the National Ethical Guidelines in Biomedical Research in Iran performed the recent study. The study protocol was approved by the ethics committee of Guilan University of Medical Sciences (IR.GUMS.REC.1396.447). All subjects provided written informed consent. The subjects could withdraw from the investigation whenever they requested.

## Results

**Figure 1:**
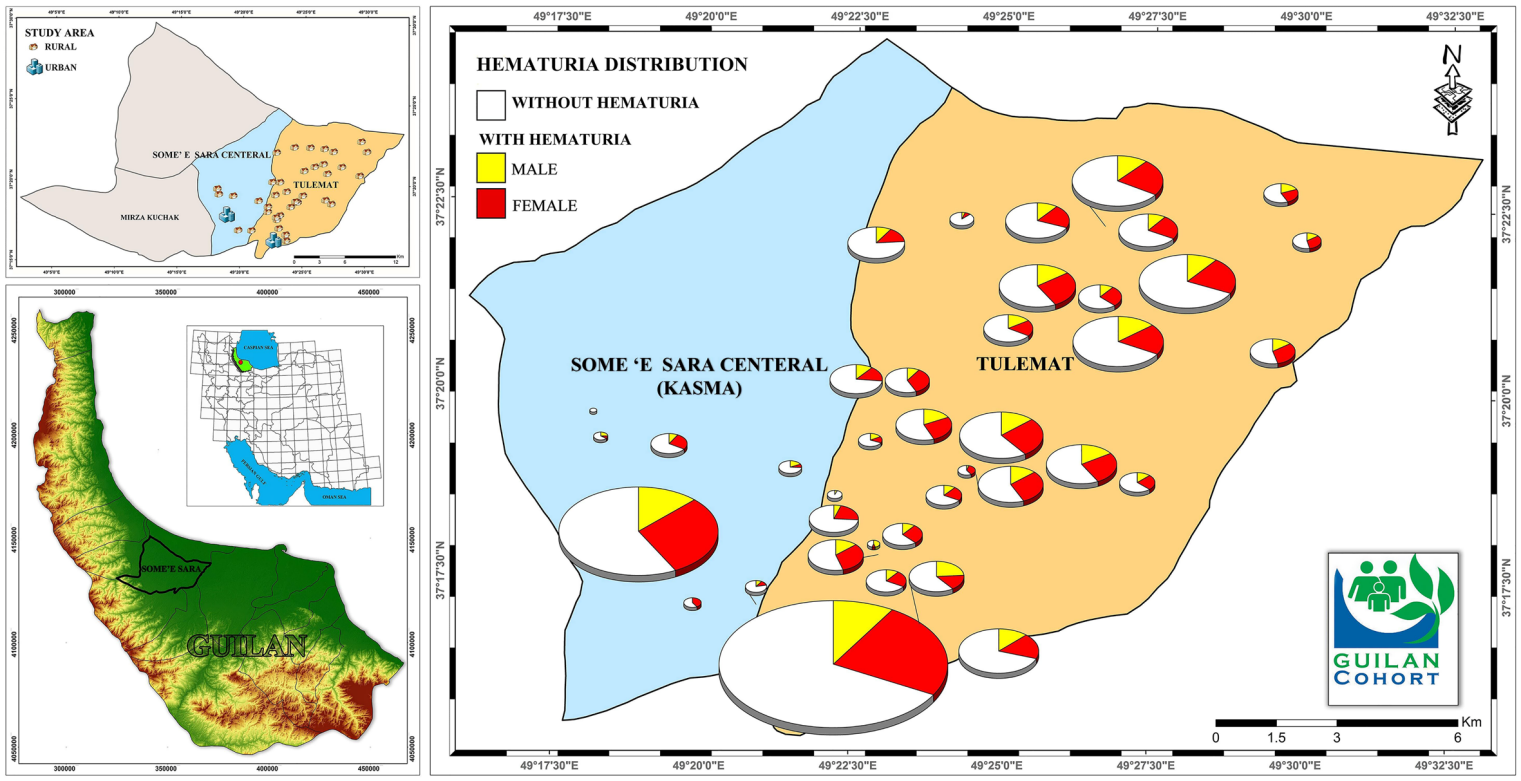
Geographic distribution of AMH prevalence in urban and rural area of Guilan cohort study. The map depicted in figure was generated by ArcGIS software (Version 10.8 for Desktop).

The demographic and clinical characteristics of the participants are presented in Table 1. The geographic distribution of AMH prevalence in the general population and different genders in urban and rural areas of the Guilan cohort study is presented in Fig. 1. The mean age of the participants was 51.52 ± 8.90 years, and 5633 (53.5%) were females. Compared to participants without AMH, participants with AMH were older, more likely to be female, more unemployed, had a low educational level, had low BMI, low WSI, high physical activity, were less likely to have diabetes and reported more consumption of alcohol and less use of opium consumption. The prevalence of AMH was 34.1% in this study, more prevalent in females than in males (38.2% vs. 29.3%).

**Table 1:** Demographic and clinical characteristics of the participants with and without AMH in the PERSIAN Guilan Cohort Study (n = 10,520).

Variable	Total	Without AMH	With AMH	P
	10,520 (%)	6935 (%)	3585 (%)	
Age (years)				< 0.001
35–44	3139 (29.8)	2162 (31.2)	977 (27.3)	
45–54	3854 (36.6)	2549 (36.8)	1305 (36.4)	
55–64	2730 (26.0)	1734 (25.0)	996 (27.8)	
> 65	797 (7.6)	490 (7.1)	307 (8.6)	
Sex				< 0.001
Male	4887 (46.5)	3453 (49.8)	1434 (40.0)	
Female	5633 (53.5)	3482 (50.2)	2151 (60.0)	
Marital status				0.251
Single	305 (2.9)	205 (3.0)	100 (2.8)	
Married	9527 (90.6)	6297 (90.8)	3230 (90.1)	
Widow	566 (5.4)	361 (5.2)	205 (5.7)	
Divorced	122 (1.1)	72 (1.0)	50 (1.4)	
Education level				< 0.001
Illiterate	1738 (16.5)	1105 (15.9)	633 (17.7)	
1–5	3312 (31.5)	2135 (30.8)	1177 (32.8)	
6–12	4832 (45.9)	3213 (46.3)	1619 (45.2)	
University	638 (6.1)	482 (7.0)	156 (4.4)	
Employment				< 0.001
Unemployed	4781 (45.4)	2986 (43.1)	1795 (50.1)	
Employed	5739 (54.6)	3949 (56.9)	1790 (49.9)	
Habitat				0.901
Urban	4613 (43.8)	3044 (43.9)	1569 (43.8)	
Rural	5907 (56.2)	3891 (56.1)	2016 (56.2)	
Wealth score index (WSI)				< 0.001
Tertile 1 (low-income)	3507 (33.3)	2236 (32.2)	1271 (35.5)	
Tertile 2 (middle-income)	3507 (33.3)	2333 (33.6)	1174 (32.7)	
Tertile 3 (high-income)	3506 (33.3)	2366 (34.1)	1140 (31.8)	
BMI (kg/m^2^)				0.005
Normal	2746 (26.1)	1813 (26.1)	933 (26.0)	
Underweight	141 (1.3)	74 (1.1)	67 (1.9)	
Overweight	4198 (39.9)	2799 (40.4)	1399 (39.0)	
Obese	3435 (32.7)	2249 (32.4)	1186 (33.1)	
MET				0.040
Tertile 1 (low)	3507 (33.3)	2362 (34.1)	1145 (31.9)	
Tertile 2 (moderate)	3507 (33.3)	2293 (33.1)	1214 (33.9)	
Tertile 3 (high)	3506 (33.3)	2280 (32.9)	1226 (34.2)	
Smoking				0.652
Non-smoker	7936 (75.4)	5241 (75.6)	2695 (75.2)	
Smoker	2584 (24.6)	1694 (24.4)	890 (24.8)	
Hookah smoking				< 0.001
No	9005 (85.6)	5875 (84.7)	3130 (87.3)	
Yes	1515 (14.4)	1060 (15.3)	455 (12.7)	
Opium consumption				< 0.001
No	9794 (93.1)	6399 (92.3)	3395 (94.7)	
Yes	726 (6.9)	536 (7.7)	190 (5.3)	
Alcohol consumption				0.001
No	9125 (86.7)	6096 (87.5)	3056 (85.2)	
Yes	1395 (13.3)	866 (12.5)	529 (14.8)	
Hypertension				0.322
No	5977 (56.8)	3964 (57.2)	2013 (56.2)	
Yes	4543 (43.2)	2971 (42.8)	1572 (43.8)	
Diabetes				< 0.001
No	7989 (75.9)	5123 (73.9)	2866 (79.9)	
Yes	2531 (24.1)	1812 (26.1)	719 (20.1)	
Kidney stone disease				0.436
No	8876 (84.4)	5865 (84.6)	3011 (84.0)	
Yes	1644 (15.6)	1070 (15.4)	574 (16.0)	

*AMH* asymptomatic microscopic hematuria, *BMI* body mass index, *METs* metabolic equivalent rates.

Simple and multiple logistic regression analysis was adopted to identify factors associated with AMH (see Table 2). Based on unadjusted analysis, the odds of AMH increased with rising age. The three older age groups (45–54, 55–64, and > 65) were significantly more likely than the youngest group (35–44) to have AMH; the OR for those aged 45–54 was 1.13 (95% CI 1.02–1.25), for those aged 55–64 was 1.27 (95% CI 1.14–1.42), and for those aged 65 and older was 1.39 (95% CI 1.18–1.63). Compared to males, females were 1.49 times more likely to have AMH than males (OR 1.49, 95% CI 1.37–1.61). Illiterate people, as well as people with primary/secondary education, were more likely to have AMH than people with university education. Employed participants reported lower AMH than unemployed participants (OR 0.75, 95% CI 0.69–0.82). Regarding WSI, participants in the second and third tertile were at significantly decreased risk for AMH compared to participants in the first. Underweight-BMI participants were at significantly higher risk for AMH than normal-BMI participants (OR 1.76, 95% CI 1.25–2.47). Regarding MET, participants in the second and third tertile were at significantly increased risk for AMH compared to participants in the first. Hookah smoking (OR 0.81, 95% CI 0.72–0.91) and opium consumption (OR 0.67, 95% CI 0.56–0.79) decreased the odds of AMH, whereas alcohol consumption increased the odds of AMH (OR 1.21, 95% CI 1.08–1.36). Diabetes decreased the likelihood of having AMH (OR 0.71, 95% CI 0.64–0.78). Other variables were not significantly associated with AMH.

**Table 2:** Logistic regression analyses for the relationship between demographic/clinical factors and AMH among adults (> 35 years old) in the PERSIAN Guilan Cohort Study (n = 10,520).

Variable	Prevalence of AMH, n (%)	Unadjusted		Adjusted	
		OR (95% CI)	*P*	OR (95% CI)	*P*
Age (years)					
35–44	977 (31.1)	1		1	
45–54	1305 (33.9)	1.13 (1.02–1.25)	0.015	1.14 (1.03–1.27)	0.012
55–64	996 (36.5)	1.27 (1.14–1.42)	< 0.001	1.35 (1.20–1.52)	< 0.001
> 65	307 (38.5)	1.39 (1.18–1.63)	< 0.001	1.46 (1.23–1.74)	< 0.001
Gender					
Male	1434 (29.3)	1		1	
Female	2151 (38.2)	1.49 (1.37–1.61)	< 0.001	2.04 (1.81–2.30)	< 0.001
Marital status					
Single	100 (32.8)	1			
Married	3230 (33.9)	1.05 (0.82–1.34)	0.685		
Widow	205 (36.2)	1.16 (0.87–1.56)	0.311		
Divorced	50 (41.2)	1.42 (0.92–2.19)	0.110		
Education level					
Illiterate	633 (36.4)	1.77 (1.44–2.17)	< 0.001	1.22 (0.98–1.52)	0.077
1–5	1177 (35.5)	1.70 (1.40–2.07)	< 0.001	1.32 (1.08–1.62)	0.008
6–12	1619 (33.5)	1.56 (1.29–1.88)	< 0.001	1.35 (1.11–1.64)	0.003
University	156 (24.5)	1		1	
Employment					
Unemployed	1795 (37.5)	1			
Employed	1790 (31.2)	0.75 (0.69–0.82)	< 0.001		
Habitat					
Urban	1569 (34.0)	1			
Rural	2016 (34.1)	1.01 (0.93–1.09)	0.901		
Wealth score index (WSI)					
Tertile 1 (low-income)	1271 (36.2)	1			
Tertile 2 (middle-income)	1174 (33.5)	0.89 (0.80–0.98)	0.015		
Tertile 3 (high-income)	1149 (32.5)	0.85 (0.77–0.94)	0.001		
BMI (kg/m^2^)					
Normal	933 (34.0)	1		1	
Underweight	67 (47.5)	1.76 (1.25–2.47)	0.001	1.65 (1.17–2.33)	0.004
Overweight	1399 (33.3)	0.97 (0.88–1.08)	0.574	0.95 (0.85–1.06)	0.341
Obese	1186 (34.5)	1.02 (0.92–1.14)	0.651	0.89 (0.79–1.00)	0.055
Physical activity (MET)					
Tertile 1 (low)	1145 (32.6)	1		1	
Tertile 2 (moderate)	1214 (34.6)	1.09 (0.99–1.21)	0.081	1.05 (0.95–1.16)	0.325
Tertile 3 (high)	1226 (35.0)	1.11 (1.00–1.22)	0.040	1.16 (1.05–1.29)	0.005
Smoking					
Non-smoker	2695 (34.0)	1		1	
Smoker	890 (34.4)	1.02 (0.93–1.12)	0.652	1.44 (1.26–1.64)	< 0.001
Hookah smoking					
No	3130 (34.8)	1			
Yes	455 (30.0)	0.81 (0.72–0.91)	< 0.001		
Opium consumption					
No	3395 (34.7)	1		1	
Yes	190 (26.2)	0.67 (0.56–0.79)	< 0.001	0.81 (0.67–0.97)	0.019
Alcohol consumption					
No	3056 (33.5)	1		1	
Yes	529 (37.9)	1.21 (1.08–1.36)	0.001	1.24 (1.09–1.42)	< 0.001
Hypertension					
No	2013 (33.7)	1			
Yes	1572 (34.6)	1.04 (0.96–1.13)	0.322		
Diabetes					
No	2866 (35.9)	1		1	
Yes	719 (28.4)	0.71 (0.64–0.78)	< 0.001	0.64 (0.58–0.71)	< 0.001
Kidney stone disease					
No	3011 (33.9)	1		1	
Yes	574 (34.9)	1.04 (0.94–1.17)	0.436	1.15 (1.03–1.29)	0.015

*AMH* asymptomatic microscopic hematuria, *BMI* body mass index, *METs* metabolic equivalent rates, *OR* odds ratio, *CI* confidence interval.

Based on multiple logistic regression analysis using backward elimination, older age, female gender, low educational level, underweight-BMI, high physical activity, smoking, alcohol consumption, and having kidney stone disease increased the likelihood of AMH, whereas opium consumption, and having diabetes decreased the likelihood of AMH.

Multiple logistic regression analyses using backward elimination were also used to identify factors associated with AMH by gender (see Table 3). In males, older age, low educational level, underweight-BMI, smoking, alcohol consumption, and having kidney stone disease increased the likelihood of AMH, whereas being resident in a rural area, obese-BMI, and having diabetes decreased the likelihood of AMH. In females, older age, residency in rural areas, and high physical activity increased the likelihood of AMH, whereas smoking and having diabetes decreased the likelihood of AMH.

**Table 3:** Logistic regression analyses for the relationship between demographic/clinical factors and hematuria for male and female adults in the PERSIAN Guilan Cohort Study (n = 10,520).

Variable	Male		Female	
	aOR (95% CI)	*P*	aOR (95% CI)	*P*
Age (years)				
35–44	1		1	
45–54	1.30 (1.10–1.52)	0.002	1.05 (0.91–1.20)	0.511
55–64	1.56 (1.31–1.87)	< 0.001	1.23 (1.06–1.43)	0.005
> 65	1.82 (1.40–2.38)	< 0.001	1.33 (1.07–1.66)	0.011
Marital status				
Single				
Married				
Widow				
Divorced				
Education level				
Illiterate	1.39 (1.01–1.91)	0.042		
1–5	1.62 (1.24–2.13)	< 0.001		
6–12	1.53 (1.19–1.98)	0.001		
University	1			
Employment				
Unemployed				
Employed				
Habitat				
Urban	1		1	
Rural	0.82 (0.72–0.94)	0.003	1.33 (1.19–1.49)	< 0.001
Wealth score index (WSI)				
Tertile 1 (low-income)				
Tertile 2 (middle-income)				
Tertile 3 (high-income)				
BMI (kg/m^2^)				
Normal	1			
Underweight	1.70 (1.15–2.53)	0.008		
Overweight	0.91 (0.79–1.05)	0.181		
Obese	0.75 (0.62–0.91)	0.004		
Physical activity (MET)				
Tertile 1 (low)			1	
Tertile 2 (moderate)			1.11 (0.98–1.26)	0.106
Tertile 3 (high)			1.35 (1.17–1.57)	< 0.001
Smoking				
Non-smoker	1		1	
Smoker	1.40 (1.22–1.61)	< 0.001	0.53 (0.30–0.96)	0.037
Hookah smoking				
No				
Yes				
Opium consumption				
No				
Yes				
Alcohol consumption				
No	1			
Yes	1.37 (1.17–1.60)	< 0.001		
Hypertension				
No				
Yes				
Diabetes				
No	1		1	
Yes	0.67 (0.57–0.79)	< 0.001	0.63 (0.56–0.72)	< 0.001
Kidney stone disease				
No	1			
Yes	1.22 (1.04–1.43)	0.017		

*AMH* asymptomatic microscopic hematuria, *BMI* body mass index, *METs* metabolic equivalent rates, *aOR* adjusted odds ratio, *CI* confidence interval.

## Discussion

The current study analyzed the prevalence of AMH and associated factors among an adult population in the north of Iran. A wide variety of genitourinary problems such as renal parenchymal disease, urolithiasis, bladder cancer, and urinary tract infection (UTI) asymptomatic: bacteriuria) can induce microscopic hematuria^1^. The overall prevalence of AMH among the studied population was about 34.1%. Past studies on different settings and age groups showed a prevalence of 2.6%^25^ and 12.6%^26^ in the cities of Isfahan and Shahreza in the central region of Iran. In addition, a cohort study with 2,421,585 participants from a managed care organization in the USA showed that 39.9% of the study population were positive for AMH in at least one urinalysis test in 2 years^27^. A recent study from Turkey suggested a prevalence of 15.9% among checkup patients^28^. In a screening program in Japan, 17.5% of participants tested positive for dipstick hematuria^29^.

Based on the analysis, older ages, and female gender are associated with AMH. The same study on Korean adults suggested being older and female as risk factors for AMH^30^. Also, past investigations found that more than 95% of people with AMH are aged older than 35 years^2^. This could explain the relatively high prevalence of AMH in this study because the population is over 35 years old. In contrast, a previous study in the general male population did not find a significant relationship between age and AMH in a Canadian masculine population^4^. Furthermore, a study in Turkey suggested that the mean age of men with AMH is significantly higher than their counterparts without AMH^28^. Older ages increase the risk of several conditions that could result in AMH, e.g., bladder cancer^31^, benign prostatic hyperplasia^32^, and UTI^33^.

Further analysis suggested that educational level is inversely associated with the risk of AMH in females but not males. This is in line with the findings of a study in a Korean population, which showed an inverse association between education level and the prevalence of AMH^30^. It has been shown that illiteracy and low educational level are significantly associated with UTI^34^ and urolithiasis^35^, which are significant causes of AMH^1^. Moreover, the analysis showed an increased risk of AMH in urban males and rural females. In contrast, a previous study in Korea found no significant association between the type of residence and the prevalence of AMH^30^.

Recent results showed a greater prevalence of AMH among underweight people. In contrast, being obese decreased the risk of AMH. Further analysis showed that this difference is limited to males. An observational study among Canadian men found no significant difference between the risk of AMH in different BMI groups^4^. In addition, a recent study among Iranian adults showed that underweight participants are at a greater risk of being diagnosed with nephrolithiasis^36^. Previous studies showed that obesity could advance prostate tissue inflammation^37^ and increase the risk of cystitis and urothelial dysplasia, which in turn may cause AMH^1^.

The analysis found a higher risk for AMH among females with higher physical activity levels. Some studies revealed that recent physical activity could cause AMH^38,39^. Past studies suggested that motorcycling, prolonged bicycling, horseback riding, and similar physical activities and exercises, as well as sexual intercourse, could increase the risk of bladder infections^40^. In addition, past analysis suggested that overexercise could increase the risk of urolithiasis, maybe through dehydration^41^.

Further analysis showed that smoking increases the risk of AMH in males but decreases it in females. Han et al. in 2013 suggested that a history of smoking decreases the risk of AMH^30^. Moreover, a retrospective study in 2019 showed no significant relationship between smoking and the prevalence of AMH in men^4^. Previous studies showed that smoking increases the risk of conditions that are associated with gross or microhematuria, such as urinary tract neoplasia^42–44^ and nephrolithiasis^45,46^. Surprisingly, our results revealed a reverse association between opium consumption and AMH. However, previous studies suggested opium consumption as a risk factor for bladder cancer, maybe through mechanisms similar to smoking^47^. Future studies with consideration of dosage, duration, and route of opium consumption are necessary to elucidate its exact effect on the prevalence of AMH. Another finding of the recent study is the increased risk of AMH in alcohol-consumer men. In line with this finding, Vartolomei et al. found that heavy alcohol consumption may increase the risk of bladder cancer in males^48^. In contrast, the results of the study among Korean adults showed a lower prevalence of AMH in alcohol consumers^30^.

In our analysis, no significant relationship between hypertension and AMH was found. Previous studies among Canadian men and Korean adults found similar results^30,49^. A study suggested that long-term hypertension may significantly increase the risk of hematuria among the group of patients with both benign prostate hyperplasia and hypertension^50^. In the present study, a past medical history of diabetes decreased the odds of AMH to around 0.6 times in both males and females. In a screening study, Kang et al. found that diabetes and male gender increased the risk of finding underlying pathology in subjects with AMH^51^. However, a previous study suggested an increased risk of AMH among diabetic patients by odds of 2.8 times^4^. Moreover, Han et al. did not find a significant relationship between the history of diabetes and AMH^30^. Polyuria is common in patients with hyperglycemia because of a glucose-induced osmotic diuresis^52^. This high volume of urinary output may result in decreased red blood cell concentration in urine samples and underestimating hematuria.

The findings of the study showed that a history of kidney stone disease increases the risk of AMH. The studies showed that nearly 90% of the cases of kidney stone disease present with either microscopic or macroscopic hematuria^53,54^. In addition, kidney stone is also known as a lifelong disease because of the high recurrence rate; Zisman AL reported that the recurrence rates of kidney stone diseases at 2, 5, 10, and 15 years were 11%, 20%, 31%, and 39%^55^. Urinary system stones may cause AMH through tissue damage^56^. Recent studies suggested a bidirectional relationship between kidney tissue inflammation and nephrolithiasis^57^. Residual renal tissue inflammation could explain the increased risk of hematuria among kidney stone formers^58^. Also, the study’s findings showed that a history of kidney stone disease increases the risk of AMH in males. A previous study in the same population showed a prevalence of 15.6% for urolithiasis in which the majority of cases were men 18.5% vs women 13.1%^59^.

The greatest strength of the recent study is the large number of subjects included, which could provide representative findings with a more accurate point estimate for the prevalence of AMH. One of the study’s limitations was that it included adults older than 35 years, and any interpretation of the study’s results should be based on this age group. Another limitation of our study was that the data gathering was based on the participants’ self-report and might have been affected by recall bias. According to the results, a significant portion of our study population is affected by AMH. This favors the cost-effectiveness of further screening programs in the Iranian population, at least in subjects with associated risk factors of AMH. Considering the lack of high-quality scientific evidence and consensus on a definite clinical guideline for AMH in our country, the results of the present study could be used to design a unit algorithm for screening and therapy of AMH. Surprisingly, being obese, opium consumption, and diabetes were associated with a lower risk of AMH. In addition, we recommend further studies exploring this relationship to delineate underlying mechanisms, which could open new insights into the pathophysiology of the conditions associated with AMH.

## Data Availability

The datasets used and/or analyzed during the current study are available from the corresponding author upon reasonable request.

## Abbreviations

RBC: Red blood cell
AMH: Asymptomatic microscopic hematuria
HPF: High power field
CKD: Chronic kidney disease
PERSIAN: Prospective Epidemiological Research Studies in IRAN
PGCS: PERSIAN Guilan cohort study
HTN: Hypertension
PCA: Principal component analysis
BMI: Body mass index
SD: Standard deviation
OR: Odds ratio
CI: Confidence interval
UTI: Urinary tract infection
MET: Metabolic equivalent of task
WSI: Wealth score index
UA: Urine analysis
